# Consumer preferences for evaluative front-of-package nutrition labels: evidence from a choice experiment in China

**DOI:** 10.3389/fnut.2025.1563341

**Published:** 2025-07-14

**Authors:** Minda Yang, Shi Min, Trung Thanh Nguyen

**Affiliations:** ^1^College of Economics and Management, Huazhong Agricultural University, Wuhan, China; ^2^Institute for Environmental Economics and World Trade, Leibniz University Hannover, Hanover, Germany

**Keywords:** evaluative FOP nutrition label, discrete choice experiment, information intervention, food choice, China

## Abstract

This study examines consumer preferences, behaviors, and willingness to pay (WTP) for evaluative front-of-package (FOP) nutrition labels in China, with a particular focus on the role of information provision in influencing consumer behavior. A choice experiment was conducted involving ham sausages with different evaluative nutrition labels, including the Health Star Rating and Nutrition Score System, among 998 participants from five representative cities in China. The choice experiment also incorporates an information intervention to assess its impact on consumers’ choice. A random parameter logit model was employed to estimate consumers preferences and WTP for the different evaluative FOP labels. The results show that consumers exhibit a higher preference for ham sausages with evaluative FOP nutrition labels compared to those without any labels. The Nutrition Score System emerges as the most preferred labeling format. Furthermore, under information intervention, consumers exhibit the highest WTP for evaluative FOP labels, with 2.47 yuan for the Health Star Rating (124% premium) and 2.77 yuan for the Nutrition Score System (138% premium). These findings suggest a substantial demand among Chinese consumers for evaluative FOP nutrition labels and underscore the importance of providing related information to enhance consumer acceptance and promote healthier food-choice behavior.

## 1 Introduction

Non-communicable diseases (NCDs) such as obesity, cardiovascular disease and type 2 diabetes have risen sharply worldwide, driven by unhealthy diets and growing consumption of ultra-processed, nutrient-deficient foods (1, 2). This trend imposes substantial economic burdens, including higher healthcare costs, reduced labor productivity and strain on public health systems (3, 4). FOP nutrition labels mitigate information asymmetry by presenting concise nutritional summaries on packaging, thereby guiding consumers toward healthier choices (5–7). Such labels have been shown to be a cost-effective tool for alleviating the socioeconomic burden of NCDs (8).

Evaluative FOP nutrition labels aim to offer a simplified, overall evaluation of a food product’s nutritional quality through metrics, enabling consumers to quickly assess the healthiness of food items (9, 10). By simplifying complex nutritional information into a more accessible format, evaluative FOP labels are especially beneficial in contexts where consumers, limited by time, attention, or knowledge, find it challenging to make fully informed decisions (11). Previous studies indicate that evaluative FOP labels further enhance consumer decision making by providing immediate feedback on the healthfulness of a food product, thus outperforming traditional BOP labels, which are often overlooked or misunderstood (12, 13). For example, De Temmerman et al. (14) found that evaluative labels significantly boosted the identification and selection of healthier products over nutrient-based labels. Similarly, Hagmann and Siegrist (15) demonstrate that Nutri-Score effectively nudges consumers toward healthier options. Moreover, Chen et al. (16) indicate that evaluative FOP labels can reduce consumers’ cognitive load when selecting food, thereby helping them choose healthier options. Consequently, evaluative FOP formats are essential for promoting healthier consumption, particularly under time or information constraints.

Consumers’ preferences for evaluative FOP labels underlie their potential health impacts. While numerous studies have investigated consumer preferences and attitudes toward FOP nutrition labels, most of this research strand has been conducted in developed countries [e.g., (17–20)]. In Australia, Health Star Ratings have been shown to steer consumers toward healthier choices through their intuitive simplicity (21). European studies confirm that Nutri-Score enhances product healthfulness identification and yields higher selection rates and WTP (22, 23). Similarly, U.S. research demonstrates that FOP labels improve consumers’ understanding of nutritional quality and inform purchasing decisions (24). Collectively, these findings suggest that consumers in developed markets generally show a positive preference for evaluative FOP labels, with substantial impact on both product choice and WTP for healthier options.

Evidence from developing economies has revealed mixed consumer responses to FOP labels. In India, some consumers value label clarity and convenience, but have low awareness and understanding of limit effectiveness (25). Chinese consumers exhibit variable comprehension and use of FOP formats, which are influenced by education, nutrition knowledge, and income (26, 27). In Brazil, while some consumers engage positively with FOP labels, others remain indifferent or skeptical because of limited nutritional literacy and unfamiliarity (28). This heterogeneity underscores the need for rigorous empirical research to identify the acceptance drivers and assess FOP label efficacy in developing markets.

The objective of this study is to investigate consumer preferences and WTP for evaluative FOP nutrition labels, using China as a case study. In particular, this study focuses on the effect of providing evaluative FOP label information on consumer acceptance. There are three reasons for choosing Chinese consumers in this study. First, China, as the world’s largest food market, plays a crucial role in shaping consumption patterns and nutritional behaviors. Encouraging Chinese consumers to adopt healthier dietary choices can advance global public health initiatives. Second, after 2013, the Chinese government required nutrition fact tables to be marked on the back of food packages. However, evaluative FOP labels have rarely been used in China. Exploring Chinese consumers’ preferences and their WTP for evaluative FOP labels can enhance the effectiveness of these labels in the global implementation of labeling systems.

Third, the significance of the research findings regarding Chinese consumers’ preferences and WTP for evaluative FOP labels pertains not only to the welfare of hundreds of millions of consumers in China but also serves as an important reference for other developing countries.

To achieve the goal, a choice experiment was conducted in an online survey involving 998 consumers across five representative cities in China (Beijing, Shenyang, Wuhan, Chengdu, and Guangzhou) in October 2020 to investigate consumers’ preferences and WTP for evaluative FOP labels. We then evaluated the impact of information intervention on consumer preferences and WTP estimates to further investigate whether information on health benefits associated with evaluative FOP labels mitigates the impact of unhealthy food choices. We further examined the sources of heterogeneity in the distribution of WTP for evaluative FOP labels by analyzing the distributional effects of these labels on consumers’ WTP across various socio-demographic characteristics. We also calculated the changes in consumer welfare across various utility specifications in the form of compensating variation (CV).

The contributions of this study are threefold. First, while prior research on FOP nutrition labels in China has predominantly focused on reductive and directive schemes, empirical evidence on consumer preferences and WTP for evaluative FOP labels remains limited (26, 27, 29). This study addresses this gap by estimating Chinese consumers’ WTP for evaluative FOP labels using a nationally representative sample, thereby expanding the limited but growing literature on FOP labeling in China. Second, existing studies often assume that respondents understand FOP symbols; however, the reality is quite different, and consumers’ comprehension of evaluative FOP labels varies (30, 31). In response, this study incorporates a brief information intervention into the choice experiment to assess how minimal educational cues influence both comprehension and valuation of evaluative FOP labels. This approach provides insight into the behavioral mechanisms by which information support can enhance the effectiveness of evaluative labeling. Third, to translate preference data into welfare-relevant metrics, CV is computed under alternative utility specifications. Although CV is widely used in discrete choice modeling (32, 33), its application in the context of FOP labeling remains rare. This study demonstrates its value for quantifying potential consumer surplus changes linked to future nutrition label policies. Taken together, these contributions advance empirical understanding of evaluative FOP labels, highlight the importance of targeted information support, and offer practical welfare estimates to inform evidence-based nutrition labeling policy in China.

The rest of this study proceeds as follows. Section 2 presents the methods, including a discrete choice modeling framework, a random parameters logit model, and a choice experimental design and data collection. Section 3 presents the estimation results and discusses the findings. Section 4 summarizes and concludes.

## 2 Methodology

### 2.1 Experimental design

To assess consumers’ preferences and WTP for evaluative FOP labels, we employed a choice experiment design that built on established frameworks from previous studies [e.g., (34–36)]. Ham sausage was selected as the experimental product for several reasons. First, China is the world’s largest ham sausage market and has a long-standing cultural preference for ham sausage. In 2019, Chinese consumption accounted for 25.4% of global ham-sausage volume and 68% of Asia-Pacific demand.^1^ This extensive consumption provides a substantial and relevant market for analyzing consumer behavior. Second, the Chinese ham-sausage category spans a wide range of quality grades, which leads to large variation in nutritional content (37). These quality differences are not always transparent to consumers, who often find it difficult to evaluate nutritional disparities across products. Such information gaps can hinder informed dietary choices and ultimately affect nutritional intake and health. By focusing on ham sausage, this study investigated Chinese consumers’ preferences and WTP for evaluative FOP labels in a major packaged-meat category.

Table 1 presents comprehensive information on the attributes and their corresponding levels employed in the choice experiment. Three levels were specified for the evaluative FOP-label attribute: Health Star Rating, Nutrition Score System, and non-evaluative FOP labels. The Health Star Rating assigns each packaged item a star rating ranging from 0.5 to 5 based on its nutritional quality, with higher ratings indicating healthier products (38). The Nutrition Score System computes an overall nutritional score on a 1–100 scale, where achieving a high score requires the product to contain below the maximum limits of fat, saturated fat, and sodium, and above the minimum thresholds of protein, dietary fiber, as well as various vitamins and minerals (39). Both schemes were designed to facilitate comparative evaluations among similar products, enabling consumers to make informed dietary choices based on standardized nutritional information. Additionally, recognizing the variability in meat content of Chinese ham sausages, which directly affects the content of high-quality protein, the choice experiment incorporated a Meat Content Claim attribute set at two levels: With Meat Content Claim and Without Meat Content Claim. This attribute accesses assess how transparency in meat content influences consumer preferences and WTP. The final attribute examined was price, reflecting the range of ham sausage prices in China at the time of the survey. The pricing levels were determined based on current market prices in supermarkets and grocery stores, as well as through consultations with consumers, ham sausage producers, and dealers to ensure relevance and accuracy. Consequently, the price attribute included three levels: a base price of 2.00 yuan per stick (50 g), a supplementary price of 2.50 yuan per stick (50 g), and a reduced price of 1.50 yuan per stick (50 g). By selecting and defining these attributes and their respective levels, the choice experiment was strategically designed to capture nuanced consumers’ preferences and WTP for various aspects of ham sausage products, including nutritional labeling, meat content transparency, and price sensitivity.

**Table 1:** Product attributes used in the choice experiment.

Attributes	Levels	Description
Evaluative FOP label	3	Health star rating
		Nutrition score system
		None
Meat contain claim	2	Yes
		No
Price (Chinese yuan)	3	1.5 yuan
		2 yuan
		2.5 yuan

Based on the selected product attributes and their levels, a full factorial design would generate 18 possible product profiles (three FOP nutrition labels × two meat contain claims × three price levels). While combining these profiles into sets of three would theoretically produce $C_{3}^{18}=816$ possible selection sets, administering such a large number is impractical in a real-world experimental setting. Moreover, extensive choice tasks can deplete participants’ cognitive resources and thus affect their judgment quality (40). To mitigate this problem, we employed a fractional factorial design that maximized D-optimality and obtained a D-efficiency score of 100%, indicating the most statistically efficient design attainable.

**Figure 1:**
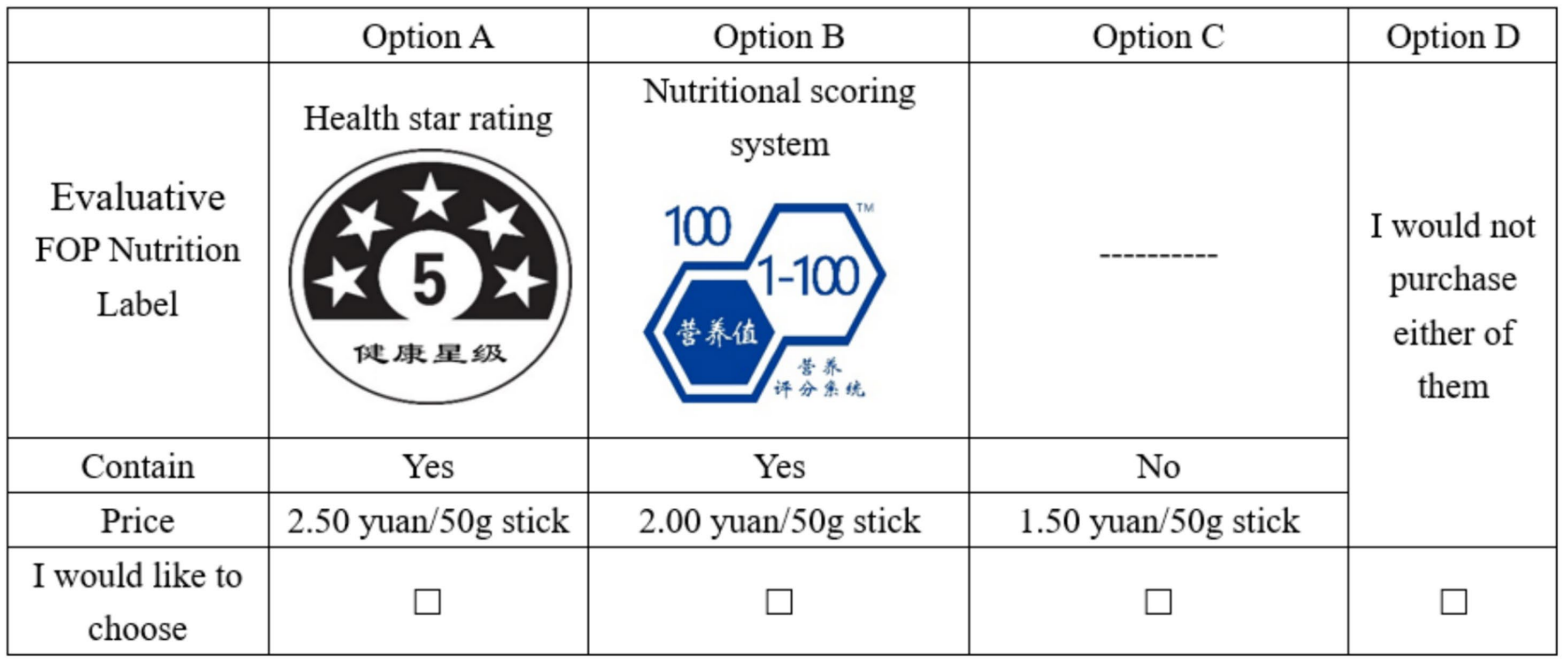
Example of a sample choice card in the choice experiment.

We generated 18 choice situations (choice cards) according to the fractional factorial design and randomly divided them into six blocks, each containing three choice situations. Consequently, each respondent was required to complete three choice sets. Each choice set presented three ham sausage options along with a fourth option of “buy nothing.” Including the no-purchase alternative replicated real market conditions and prevented respondents from feeling obliged to select an option they would not normally buy, thereby enhancing data validity and reliability (41). The case of a choice card is presented in Figure 1. Prior to the choice experiment, participants were provided with a brief presentation explaining the evaluative FOP labels and meat content claims to ensure they fully understood these attributes. Additionally, we implemented a cheap talk script to mitigate hypothetical bias (42), aiming to align consumers’ stated WTP for ham sausage more closely with their actual purchasing behavior (43).

To evaluate the effect of introducing evaluative FOP labels on consumers’ preferences and WTP for ham sausage, we conducted a randomized information intervention prior to the choice experiment. Participants were randomly assigned to either an information intervention group or a control group. The information intervention group received introductory information detailing the positive role of evaluative FOP labels in guiding healthier consumer choices and improving their nutritional awareness. In contrast, the control group did not receive any additional information before the choice experiments. This design allowed us to isolate the impact of information exposure on consumers’ preferences and WTP. The specific content of the information intervention is presented in Appendix Figure A1.

### 2.2 Econometric models

According to the random utility theory proposed by Lancaster (44), the utility $U_{\mathrm{imt}}$ that consumer *i* derives from choosing alternative *m* in choice situation *t* is composed of a deterministic component $V_{\mathrm{imt}}$ and a random error term $\varepsilon_{\mathrm{imt}}$ :


(1) $$U_{\mathrm{imt}}=V_{\mathrm{imt}}+\varepsilon_{\mathrm{imt}}$$

The deterministic component $V_{\mathrm{imt}}$ captures the observable part of the utility and is specified as the linear combination of the attributes of the alternative and the consumer’s preference parameters as in Equation 2:


(2) $$V_{\mathrm{imt}}=\beta_{i}^{\prime }X_{\mathrm{imt}}$$

where $\beta_{i}$ is a vector of preference parameters specific to consumer *i*, reflecting the weights that the consumer assigns to each attribute of the product. The vector $X_{\mathrm{imt}}$ represents the attributes of alternative *m* as perceived by consumer *i* in choice situation *t*.

Assuming that the error term $\varepsilon_{\mathrm{imt}}$ is independently and identically distributed (IID) with a Type I extreme value distribution, and that the independence of irrelevant alternatives (IIA) property holds, we can apply the conditional logit (CL) model. Under these assumptions, the probability that consumer *i* chooses alternative *m* in choice situation *t* is modeled as in Equation 3:


(3) $$P_{\mathrm{imt}}=\int \frac{\exp(V_{\mathrm{imt}})}{\sum_{j=1}^{J}\exp(V_{\mathrm{ijt}})}$$

where *J* represents the total number of alternatives in the choice set. The CL model was straightforward to estimate using maximum likelihood estimation (45). However, the IIA assumption can be restrictive because it implies that the relative odds of choosing between any two alternatives are unaffected by the presence or characteristics of other alternatives.

To address this limitation, Train (46) developed the random parameter logit (RPL) model, which offers greater flexibility by relaxing the IIA assumption and incorporating preference heterogeneity among consumers. The RPL model also accounts for correlations between multiple choice observations made by each respondent (47). In the RPL model, the preference parameters $\beta_{i}$ are treated as random variables drawn from a specified distribution characterized by a mean vector $\beta_{0}$ and a covariance matrix G as in Equation 4:


(4) $$\beta_{i}\sim f(\beta_{0},G)$$

Given this specification, the choice probability identified by the RPL model is expressed as an integral over the distribution of β as in Equation 5:


(5) $$P_{\mathrm{imt}}=\int (\frac{\exp(\beta \prime X_{\mathrm{imt}})}{\sum_{j=1}^{J}\exp(\beta \prime X_{\mathrm{ijt}})})f(\beta |,\beta_{0}|,G)\mathrm{d\beta}$$

where the coefficients in vector β are defined as random variables following density function f(β) . With the probability evaluated over a range of possible values of $\beta_{i}$ and the absence of a closed-form solution, the approach of approximating the likelihood function with the simulated maximum likelihood was applied to the model (48).

To examine the potential interaction effects between key attributes in the choice experiment and demographic variables, we extended the random utility model in Equation 1 by incorporating the interaction terms as in Equation 6:


(6) $$\begin{array}{l} U_{\mathrm{imt}}=\beta_{0}\beta_{1}\text{Pric}e_{\mathrm{imt}}+\beta_{2}\text{HealthSta}r_{\mathrm{imt}}+\beta_{4}\text{Clai}m_{\mathrm{imt}}\\ \;+\beta_{5}(\text{HealthSta}r_{\mathrm{imt}}\times Z_{i})+\beta_{6}(\text{NutritionScor}e_{\mathrm{imt}}\times Z_{i})\\ \begin{array}{l} \;+\beta_{7}(\text{Clai}m_{\mathrm{imt}}\times Z_{i})+\beta_{8}(\text{Pric}e_{\mathrm{imt}}\times Z_{i})\\ +\beta_{9}(\text{None}\times Z_{i})+\varepsilon_{\mathrm{imt}} \end{array} \end{array}$$

where $\text{Pric}e_{\mathrm{imt}}$ is the price variable, represented by the experimentally designed price levels. *None* is an alternative-specific constant representing the no-buy option. $\text{Health}\;\mathrm{Sta}r_{\mathrm{imt}}$ and $\text{Nutrition Scor}e_{\mathrm{imt}}$ are categorical variables indicating the presence of the Health Star Rating and Nutrition Score System labels on the ham sausage products, respectively, with the absence of an evaluative FOP label serving as the base category. $\text{Clai}m_{\mathrm{imt}}$ is a categorical variable for the meat content claim of the ham sausage, where no meat content claim is the base category. $Z_{i}$ represents demographic variables such as age, income level, and other relevant consumer characteristics. Following Shi et al. (49), we assumed that the coefficient for price is fixed across individuals, whereas the coefficients for the other attributes are random and follow a joint normal distribution.

The WTP measures for an attribute *k* is approximated as the coefficient estimate for the attribute divided by the negative marginal utility of price as in Equation 7:


(7) $${\mathrm{WTP}}_{k}=\frac{-\beta_{k}}{\beta_{p}}$$

where $\beta_{k}$ is the coefficient of nonprice attribute *k* and $\beta_{p}$ is the estimated price coefficient. We employed dummy coding for non-price attributes to facilitate the interpretation of coefficients relative to the base categories. The 95% confidence intervals for the WTP estimates are calculated using the delta method, which is considered accurate when the data are well-conditioned (50). All models were estimated to use Stata 17.0, utilizing 500 Halton draws for the simulations to approximate the integrals in the RPL model. The estimation accounts for the panel structure of the data, recognizing that each respondent provides multiple observations across different choice situations. This methodological approach enhances the reliability of the estimates by appropriately handling the repeated measures nature of the data.

### 2.3 Survey and data

The data for this study were collected through an online survey conducted among Chinese urban consumers in October 2020. The survey was implemented on Credamo, a reputable research platform widely recognized in China for its robust data collection capabilities. Credamo’s sample library comprises over 3 million participants from all provincial-level administrative regions across the country, ensuring extensive geographic coverage and diversity in the sample. To maintain the authenticity and reliability of the data, Credamo required all participants to complete real-name authentication and mobile number verification before being included in its sample library. This stringent verification process minimizes the risk of fraudulent responses and enhances the data integrity. Additionally, Credamo employs intelligent human-machine verification, identity verification, and IP address restrictions to prevent participants from completing the questionnaire multiple times, thereby reducing potential biases associated with duplicate responses.

For this study, questionnaires were randomly distributed to participants based on specific criteria such as region, ensuring randomness and representativeness of the data collected. Credamo also implemented advanced data quality control measures, including restrictions on participants’ credit scores, response history, and the devices used for answering surveys. These measures were designed to secure a high-quality respondent pool and maintain the overall quality of the data. Credamo’s platform is widely utilized by academic institutions and researchers, providing services to over 3,000 universities worldwide, including prestigious institutions such as the Massachusetts Institute of Technology, Columbia University, and Peking University. Its widespread adoption and reputation in the academic community are indicative of the credibility and reliability of the data collected through its platform (51, 52).

The survey was conducted in five major Chinese cities: Beijing; Shenyang in Liaoning Province; Wuhan in Hubei Province; Guangzhou in Guangdong Province; and Chengdu in Sichuan Province. These cities were strategically selected to cover northern, central, southern, and western regions and to encompass a range of urban economic development and retail infrastructure, thereby providing a comprehensive view of urban consumers’ preferences and WTP for evaluative FOP nutrition labels. The geographical distribution of these cities is illustrated in Appendix Figure A2. All respondents resided in economically advanced urban areas, where households typically enjoy higher disposable incomes, greater exposure to packaged foods, and stronger nutrition knowledge than their rural areas (53). Consequently, the promotion and adoption of evaluative FOP labels are likely to be more successful in Chinese cities than in rural regions.

In each city, 200 participants were randomly selected, resulting in a total of 1,000 distributed questionnaires. The online survey yielded 998 valid responses, indicating a high response rate and enhancing the reliability of the data collected. The survey instrument comprised of three sections. The first section collected information on consumers’ general ham sausage consumption habits and food shopping behaviors. The second section included a discrete choice experiment to estimate consumers’ preferences and WTP for the specified product attributes. The third section recorded sociodemographic information, including gender, age, household size, education level, and annual household income.

Table 2 presents the sociodemographic characteristics of the survey participants. Female respondents constituted slightly more than half of the sample were female, accounting for approximately 50.1%. Regarding educational attainment, the majority of respondents (73.4%) reported having completed some college, technical school, or holding an associate’s degree. In terms of monthly income, most participants (36.3%) earned between ¥5,000 and ¥8,000. The average household size was four members, and 56.8% of the respondents indicated that they had a child or children under the age of 16 living in their household. Additionally, 35.4% of participants reported that there were obese individuals among their acquaintances or family members. The sample was nearly evenly divided between the intervention group and the control group, with 505 subjects (50.6%) in the intervention group and 493 subjects (49.4%) in the control group. Results from the t-tests indicate that there were no significant differences in sociodemographic characteristics between the control and intervention groups, suggesting that randomization was effective.

**Table 2:** Summary statistics of sample.

Variables	Coding and description	Total (*N* = 998)		Without information (*N* = 505)		With information (*N* = 493)		Mean Diff
		Mean	Standard deviation	Mean	Standard deviation	Mean	Standard deviation	
Age	Years	28.663	8.422	28.869	8.384	28.452	8.463	0.417
Gender	1 = male; 0 = female	0.466	0.499	0.477	0.500	0.454	0.498	0.023
Married	1 = Yes; 0 = No	0.473	0.500	0.475	0.500	0.471	0.500	0.005
Education	Education with college or advanced degree 1 = Yes; 0 = No	0.734	0.442	0.750	0.433	0.718	0.450	0.032
Household size	Number of people in household	4.055	1.170	4.103	1.157	4.006	1.182	0.097
Children	Whether there are children under 16 in the family 1 = Yes; 0 = No	0.568	0.496	0.586	0.493	0.550	0.498	0.036
Low income	Household monthly income per capita (thousand yuan) (1: income≤5; 0: otherwise)	0.315	0.464	0.285	0.452	0.345	0.476	−0.060^**^
Middle income	Household monthly income per capita (thousand yuan) (1: 5 < income≤8; 0: otherwise)	0.363	0.481	0.382	0.486	0.343	0.475	0.039
High income	Household monthly income per capita (thousand yuan) (1: income>8; 0: otherwise)	0.323	0.468	0.333	0.472	0.312	0.464	0.020
Obesity	Are there any obese people you know? (1 = Yes; 0 = No)	0.354	0.478	0.362	0.481	0.345	0.476	0.018
Beijing	1 = Yes; 0 = No	0.199	0.400	0.189	0.392	0.210	0.408	0.021
Shenyang	1 = Yes; 0 = No	0.200	0.401	0.215	0.411	0.186	0.390	−0.029
Wuhan	1 = Yes; 0 = No	0.200	0.401	0.215	0.411	0.186	0.390	−0.029
Guangzhou	1 = Yes; 0 = No	0.199	0.400	0.185	0.388	0.214	0.410	0.029
Chengdu	1 = Yes; 0 = No	0.200	0.401	0.197	0.398	0.204	0.403	0.007

** *p* < 0.05.

## 3 Results and discussion

### 3.1 Estimation results of RPL models

We estimated the RPL model separately for the pooled group, information intervention group, and control group, incorporating the interaction effects in each formulation. The estimation results are summarized in Table 3. Across all models, the price coefficient is negative and statistically significant, indicating that increasing the price of ham sausage reduces utility and, consequently, the likelihood of selection. Regarding the evaluative FOP labels, with “no evaluative FOP label” as the baseline, the mean part-worth utility estimates for both Health Star Rating and Nutrition Score System are positive and significant. This result suggests that ham sausage products carrying an evaluative FOP label are more likely to be chosen compared to those without any label. Additionally, the significant standard deviation estimates for both labels indicate high variability in consumer preferences for these attributes, indicating heterogeneous valuations among individuals. With “no meat content claim” as the reference category, the positive and significant mean coefficient for the Meat Content Claim indicates that ham sausages displaying this claim generate higher consumer utility, which translates into a greater likelihood of selection. However, most interactions between the attributes and demographic variables are not significant, indicating that these variables did not substantially explain the observed preference heterogeneity. Notably, the interaction between the Nutrition Score System and Age is negative and significant, implying that older consumers are less likely to prefer products with this label, possibly because of a greater resistance to adopting new information formats. Conversely, the interactions between Education Level and both evaluative FOP labels are significantly positive, suggesting that consumers with higher educational attainment are more receptive to new labeling systems, which significantly influences their choice behavior (54).

**Table 3:** Estimation results of RPL models.

Variables	Pooled	Information	
		No	Yes
Price	−1.416^***^	−1.697^***^	−1.143^***^
	(0.325)	(0.490)	(0.427)
Star	2.204^***^	2.450^**^	2.093^**^
	(0.780)	(1.159)	(1.044)
Score	2.632^***^	2.964^***^	2.423^**^
	(0.793)	(1.143)	(1.076)
Contain	0.376	0.256	0.451
	(0.258)	(0.422)	(0.320)
No choice	−3.409^***^	−5.188^***^	−2.116
	(1.236)	(1.772)	(1.628)
Age*Star	0.002	0.008	−0.002
	(0.021)	(0.028)	(0.030)
Age*Score	−0.035^*^	−0.036	−0.032
	(0.020)	(0.029)	(0.029)
Age*Contain	0.001	0.003	0.001
	(0.007)	(0.010)	(0.009)
Age*Price	0.035^***^	0.043^***^	0.028^**^
	(0.008)	(0.012)	(0.011)
Age*No choice	0.050	0.090^**^	0.020
	(0.034)	(0.044)	(0.049)
Gender*Star	−0.480	−0.363	−0.629
	(0.295)	(0.445)	(0.404)
Gender*Score	−0.540^*^	−0.314	−0.803^*^
	(0.296)	(0.441)	(0.412)
Gender*Contain	−0.284^***^	−0.289^*^	−0.265^*^
	(0.110)	(0.167)	(0.145)
Gender*Price	0.213^*^	0.248	0.195
	(0.125)	(0.188)	(0.169)
Gender*No choice	−0.708	−0.761	−0.588
	(0.442)	(0.628)	(0.621)
Edu*Star	0.745^**^	0.723	0.803^*^
	(0.312)	(0.489)	(0.417)
Edu*Score	0.685^**^	0.716	0.700
	(0.316)	(0.465)	(0.437)
Edu*Contain	0.223^*^	0.457^**^	0.018
	(0.125)	(0.195)	(0.166)
Edu*Price	−0.013	0.074	−0.097
	(0.147)	(0.224)	(0.194)
Edu*No choice	0.890^*^	1.650^**^	0.358
	(0.501)	(0.687)	(0.704)
Mid income*Star	−0.335	−0.307	−0.506
	(0.361)	(0.562)	(0.476)
Mid income*Score	−0.220	−0.258	−0.284
	(0.361)	(0.542)	(0.494)
Mid income*Contain	−0.017	−0.034	−0.050
	(0.137)	(0.212)	(0.179)
Mid income*Price	−0.105	−0.489^**^	0.180
	(0.154)	(0.232)	(0.207)
Mid income*No choice	−0.684	−0.836	−0.961
	(0.542)	(0.775)	(0.774)
High income*Star	0.179	0.117	0.078
	(0.398)	(0.614)	(0.521)
High income*Score	0.240	0.112	0.230
	(0.391)	(0.584)	(0.527)
High income*Contain	−0.004	−0.202	0.162
	(0.145)	(0.223)	(0.187)
High income*Price	−0.060	−0.240	0.039
	(0.154)	(0.232)	(0.207)
High income*No choice	−0.550	−0.815	−0.557
	(0.582)	(0.852)	(0.781)
Work*Star	−0.190	−0.127	−0.311
	(0.318)	(0.493)	(0.430)
Work*Score	−0.206	−0.059	−0.400
	(0.319)	(0.484)	(0.441)
Work*Contain	−0.089	0.048	−0.217
	(0.118)	(0.178)	(0.159)
Work*Price	0.274^**^	0.640^***^	−0.037
	(0.139)	(0.200)	(0.196)
Work*No choice	0.502	1.628^**^	−0.487
	(0.477)	(0.700)	(0.672)
Province Control	Yes	Yes	Yes
SD			
Star	0.926^***^	1.108^***^	0.692^***^
	(0.132)	(0.177)	(0.239)
Score	0.648^***^	0.526	0.727^***^
	(0.180)	(0.354)	(0.232)
Contain	0.795^***^	0.941^***^	0.636^***^
	(0.099)	(0.135)	(0.155)
No choice	1.857^***^	1.762^***^	2.045^***^
	(0.246)	(0.334)	(0.346)
*AIC*	6,477.430	3,236.664	3,299.849
*BIC*	6,913.479	3,632.522	3,694.288
Log pseudo likelihood	−3,179.715	−1,559.332	−1,590.924
Wald Chi2	587.53^***^	334.32^***^	295.72^***^
*N*	11,976	6,060	5,916

Robust standard errors in parentheses. ^*^p < 0.10, ^**^p < 0.05, ^***^p < 0.01.

The pronounced consumer preference for evaluative FOP labels is consistent with empirical findings from other studies. Studies in Australia reported positive and significant utility gains for the Health Star Rating, confirming that a simple star-based summary increased product attractiveness (21, 55). Similar studies in Belgium and Germany showed that Nutri-Score yielded the largest part-worth utilities among competing FOP signals, mirroring the strong positive coefficient we observed for the Nutrition Score System (22, 23). Preference models in the United States likewise indicated that interpretive labels raised choice probabilities relative to nutrient tables (24). By contrast, a recent study in India found smaller or insignificant utility shifts and attributed the weaker response to limited label familiarity and lower nutrition literacy (25).

### 3.2 WTP for ham sausages with different attributes

**Figure 2:**
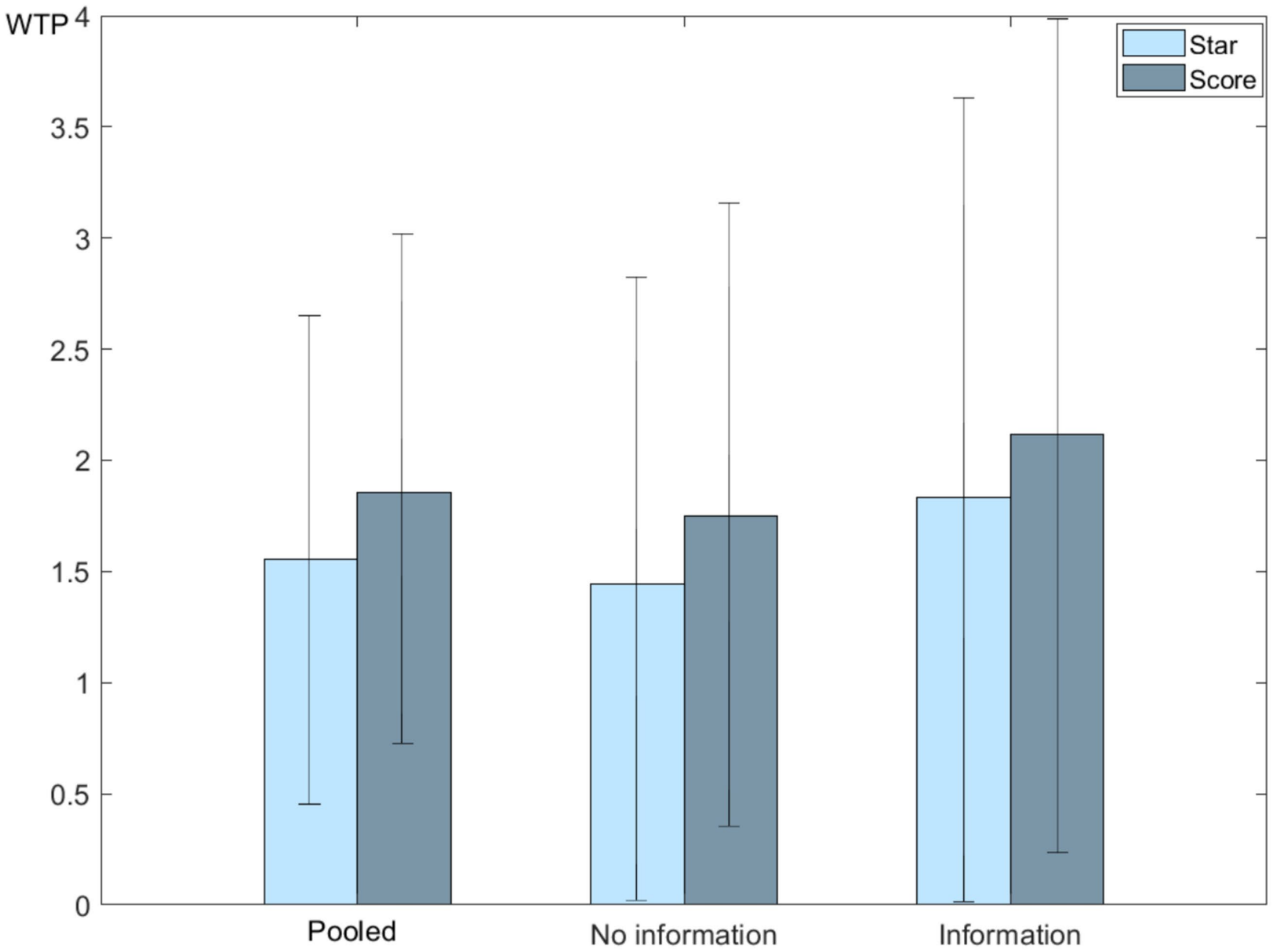
WTP of evaluative FOP labels.

Figure 2 illustrates the mean WTP estimates for the information intervention and control groups, with detailed values and confidence intervals appear in Appendix Table A1. Consumers consistently prefer the Nutrition Score System to the Health Star Rating, reflecting a clearer assessment of nutritional quality. Relative to a 2.00 yuan benchmark per 50 g stick, the intervention group’s mean WTP is 2.474 yuan for the Health Star Rating, corresponding to a 124% premium, and 2.767 yuan for the Nutrition Score System, corresponding to a 138% premium, compared with products without any evaluative FOP label. In the control group, the mean WTP is 1.711 yuan for the Health Star Rating, corresponding to an 86% premium, and 2.229 yuan for the Nutrition Score System, corresponding to an 111% premium. The full sample shows the mean WTP of 2.058 yuan for the Health Star Rating, corresponding to a 103% premium, and 2.480 yuan for the Nutrition Score System, corresponding to a 124% premium. Moreover, the mean WTPs for both evaluative FOP labels are highest under information intervention, intermediate in the full sample, and lowest in the control group, confirming that explanatory information significantly enhances the consumer valuation of evaluative FOP labels (7).

The positive WTP for evaluative FOP labels corroborates extant global research indicating that consumers are willing to pay price premiums for products displaying interpretive nutrition information. This result converges with observations from several European markets, where front-of-pack label schemes such as Nutri-Score have gained widespread consumer acceptance and strengthen purchase intent (22, 56). North American studies likewise report that certified health-related claims command sizable premiums, underscoring the economic value that shoppers assign to clear nutritional cues (57).

Table 4 presents the heterogeneity analysis of WTP estimates across different demographic groups for the Health Star Rating, Nutrition Score System, and Meat Content Claim attributes, divided into each group. For Health Star Rating, the interaction with education is positive and statistically significant across the pooled group and the information intervention group, indicating that higher education is associated with a greater WTP for products with the Health Star Rating. In the pooled sample, highly educated consumers are willing to pay 2.082 yuan, corresponding to a 104% premium over the 2.00 yuan benchmark; in the information group, their WTP rises to 2.534 yuan, corresponding to a 127% premium, indicating that education amplifies the effect of information intervention. For the Nutrition Score System, interactions with age, gender, and education are included. The interaction between education and the Nutrition Score System is positive and significant in all samples, yielding a WTP of 2.341 yuan in the pooled group, corresponding to a 117% premium, which confirms the stronger preference among more educated consumers. The interaction between age and the Nutrition Score System is also significant, with older consumers showing a WTP of 1.834 yuan, corresponding to a 92% premium, suggesting a modestly higher valuation for this label among older respondents. Finally, for the Meat Content Claim, the interactions with gender and education are mostly insignificant, except for a positive WTP for education in both the pooled and “No information” groups, suggesting that the meat content claim attribute has limited differentiation based on gender and education levels compared to evaluative labels. To sum up, these results support previous studies, indicating that educational level significantly influences consumers’ preferences for nutritional information (58, 59). Meanwhile, consumers also exhibit heterogeneity in their WTP for the Nutrition Score System based on age, which is consistent with Elia and Stratton (60).

**Table 4:** Heterogeneity analysis of WTP estimation.

Variables	Pooled		Information			
			No		Yes	
	WTP	90% CI	WTP	90% CI	WTP	90% CI
Star						
Edu*Star	2.082	[0.936, 3.227]			2.534	[0.592, 4.476]
	(0.696)				(1.181)	
Score						
Age*Score	1.834	[0.705, 2.962]				
	(0.686)					
Gender*Score	1.477	[0.400, 2.553]			1.417	[−0.264, 3.098]
	(0.654)				(1.022)	
Edu*Score	2.341	[1.148, 3.535]				
	(0.726)					
Contain						
Gender*Contain	0.065	[−0.245, 0.375]	−0.020	[−0.432, 0.393]	0.163	[−0.339, 0.664]
	(0.189)		(0.251)		(0.305)	
Edu*Contain	0.423	[0.096, 0.749]	0.420	[0.007, 0.832]		
	(0.199)		(0.251)			

### 3.3 Robustness check

To confirm the robustness of our main findings, we further employed a Generalized Multinomial Logit II (G-MNL II) model as an alternative model to re-examine consumers’ preferences and WTP for ham sausage with different evaluative FOP nutrition labels. The Price coefficient is consistently negative and statistically significant in all models, indicating that higher prices reduce utility, with the strongest negative effect observed in the “No information” group, suggesting heightened price sensitivity among uninformed consumers. Table 5 shows that the impacts of all proposed attributes, including the evaluative FOP labels and meat contain claim, are significant at least at the 5% level, which is consistent with the results presented in Table 3. Specifically, Nutrition Score System has a higher estimated effect than Health Star Rating across all groups, highlighting its greater impact on consumer choices. The Meat Content Claim coefficient is positive but not consistently significant across groups, suggesting a less pronounced effect on consumer preferences compared to evaluative FOP labels. Interactions between demographic variables and product attributes reveal mixed significance, with Education showing positive and significant interaction effects for the Health Star Rating and Nutrition Score System, indicating that more educated consumers are more likely to value these evaluative FOP labels. Meanwhile, the interaction effects involving age and gender are largely insignificant, suggesting limited differentiation in preferences based on these demographic factors. Notably, the inclusion of demographic interactions adds further insight into the heterogeneity of consumer preferences, with education being the most influential demographic in shaping preferences for nutritional information. Overall, the G-MNL II results align with earlier findings, providing robustness to the observed effects of evaluative FOP labels and prices on consumer choice behavior.

**Table 5:** Estimation results of GMNL-II models.

Variables	Pooled	Information	
		No	Yes
Price	−1.224^***^	−1.497^***^	−0.956^***^
	(0.274)	(0.412)	(0.370)
Star	2.109^***^	2.219^**^	2.020^**^
	(0.725)	(1.034)	(0.997)
Score	2.454^***^	2.701^***^	2.311^**^
	(0.741)	(1.033)	(1.026)
Contain	0.336	0.223	0.430
	(0.220)	(0.328)	(0.282)
No choice	−1.924^*^	−3.462^**^	−0.746
	(1.051)	(1.453)	(1.397)
Age*Star	−0.002	0.004	−0.003
	(0.019)	(0.025)	(0.028)
Age*Score	−0.032^*^	−0.033	−0.029
	(0.019)	(0.027)	(0.027)
Age*Contain	−0.000	0.001	−0.000
	(0.006)	(0.008)	(0.008)
Age*Price	0.031^***^	0.039^***^	0.025^**^
	(0.007)	(0.010)	(0.010)
Age*No choice	0.045	0.079^**^	0.021
	(0.029)	(0.038)	(0.043)
Gender*Star	−0.428	−0.286	−0.606
	(0.279)	(0.425)	(0.386)
Gender*Score	−0.472^*^	−0.220	−0.762^*^
	(0.282)	(0.427)	(0.398)
Gender*Contain	−0.231^**^	−0.277^**^	−0.234^*^
	(0.092)	(0.134)	(0.128)
Gender*Price	0.203^*^	0.228	0.180
	(0.107)	(0.154)	(0.151)
Gender*No choice	−0.620	−0.590	−0.668
	(0.382)	(0.548)	(0.550)
Edu*Star	0.679^**^	0.725	0.753^*^
	(0.293)	(0.445)	(0.404)
Edu*Score	0.646^**^	0.720	0.680
	(0.300)	(0.438)	(0.423)
Edu*Contain	0.182^*^	0.374^**^	0.008
	(0.105)	(0.148)	(0.143)
Edu*Price	−0.016	0.023	−0.115
	(0.126)	(0.188)	(0.171)
Edu*No choice	0.762^*^	1.187^*^	0.377
	(0.447)	(0.608)	(0.620)
Mid income*Star	−0.280	−0.332	−0.478
	(0.341)	(0.524)	(0.456)
Mid income*Score	−0.184	−0.314	−0.273
	(0.345)	(0.519)	(0.474)
Mid income*Contain	−0.003	0.003	−0.049
	(0.116)	(0.167)	(0.159)
Mid income*Price	−0.101	−0.364^*^	0.142
	(0.132)	(0.188)	(0.184)
Mid income*No choice	−0.648	−0.783	−0.816
	(0.473)	(0.675)	(0.685)
High income*Star	0.229	0.230	0.060
	(0.379)	(0.579)	(0.496)
High income*Score	0.290	0.244	0.204
	(0.376)	(0.563)	(0.503)
High income*Contain	0.008	−0.202	0.147
	(0.124)	(0.177)	(0.164)
High income*Price	−0.094	−0.224	−0.001
	(0.132)	(0.190)	(0.183)
High income*No choice	−0.432	−0.656	−0.415
	(0.511)	(0.756)	(0.678)
Work*Star	−0.137	−0.108	−0.299
	(0.298)	(0.469)	(0.406)
Work*Score	−0.166	−0.094	−0.372
	(0.302)	(0.469)	(0.417)
Work*Contain	−0.065	0.068	−0.169
	(0.100)	(0.141)	(0.139)
Work*Price	0.218^*^	0.537^***^	−0.057
	(0.118)	(0.162)	(0.173)
Work*No choice	0.387	1.398^**^	−0.519
	(0.418)	(0.615)	(0.595)
Province Control	Yes	Yes	Yes
SD			
Star	0.028	0.034	−0.013
	(0.045)	(0.069)	(0.063)
Score	−0.014	0.037	−0.019
	(0.046)	(0.069)	(0.063)
Contain	0.014	0.066	0.057
	(0.044)	(0.064)	(0.064)
No choice	−0.104	0.244	−0.295^**^
	(0.124)	(0.150)	(0.142)
*AIC*	6,628.811	3,312.470	3,360.473
*BIC*	7,072.250	3,715.038	3,761.598
Log pseudo likelihood	−3,252.405	−1,596.235	−1,620.236
Wald Chi2	917.66^***^	550.35^***^	455.45^***^
*N*	11,976	6,060	5,916

Robust standard errors in parentheses. ^*^p < 0.10, ^**^p < 0.05, ^***^p < 0.01.

### 3.4 Consumer welfare measurement

CV is a measure to quantify the monetary equivalent of a change in consumer utility, capturing the value that consumers place on improvements in product attributes changes (32, 61). In this study, CV was used to evaluate the welfare impact of introducing evaluative FOP labels, such as the Health Star Rating and Nutrition Score System, on consumer preferences for ham sausage. The compensating variation is calculated as follows:


(8) $$\begin{array}{l} E\{\max_{j\in J^{0}}[U_{j}(p_{j}^{0},q_{j}^{0},\varepsilon_{j})]\}=\\ E\{\max_{j\in J^{1}}[U_{j}((p_{j}^{1}+\mathrm{CV}),q_{j}^{1},\varepsilon_{j})]\} \end{array}$$

where $p_{j}^{0}$ and $q_{j}^{0}$ represent the price and attributes of the alternatives before the change, respectively; $p_{j}^{1}$ and $q_{j}^{1}$ represent the price and attributes of the alternatives after the change, respectively. Any numerical process that satisfies Equation 8 yields a CV value.

Table 6 summarizes the welfare impacts under four different scenarios involving simulated products with and without evaluative labels, based on CV estimates for the pooled sample, information intervention group, and control group. The results indicate that introducing the Health Star Rating resulted in a positive welfare effect across all groups, with the information intervention group experiencing the greatest welfare increase. Introducing the Nutrition Score System yields even higher welfare gains, highlighting that consumers value the detailed nutritional information provided by this label more than the Health Star Rating does. The welfare change when transitioning from a Health Star Rating to a Nutrition Score System is positive, but relatively modest, indicating that while consumers prefer the Nutrition Score System, the additional welfare benefit compared to the Health Star Rating alone is limited. Finally, introducing both evaluative labels results in substantial welfare improvements, with the information intervention group experiencing the greatest welfare gains, suggesting that consumer education further enhances the perceived value of the labeling systems. These findings demonstrate that the use of evaluative FOP labels significantly enhances consumer welfare, with the Nutrition Score System showing the highest potential for improving welfare, especially when coupled with consumer information interventions. This welfare analysis underscores the importance of effective label design and information provision for maximizing consumer benefits.

**Table 6:** Consumer welfare with simulated products.

Scenarios	Pooled	Information	
		No	Yes
S1 (No label to Health Star rating)	1.535	1.436	1.770
S2 (No label to Nutrition Score system)	1.828	1.735	2.030
S3 (Health Star rating to Nutrition Score system)	0.272	0.295	0.186
S4 (No label to Health Star rating and Nutrition Score system)	1.663	1.614	1.746

## 4 Summary and conclusion

Evaluative FOP nutrition labels play an important role in guiding consumers’ food choice behaviors by providing clear and accessible nutritional information, which helps consumers make healthier decisions and adopt positive eating habits (19, 21, 22). This study investigated consumer preferences and WTP for evaluative FOP nutrition labels on ham sausage products in China, specifically the Health Star Rating and Nutrition Score System. Using data collected from an online survey of urban Chinese consumers, we employed the RPL and G-MNL II models to assess the effects of FOP labels on consumer choice behavior. We further analyzed the impact of information intervention on consumer preference and estimated the CV to quantify changes in consumer welfare associated with different labeling scenarios. The results demonstrate that the presence of evaluative FOP labels significantly enhances consumer preferences for ham sausage products. Among the labels, the Nutrition Score System consistently yielded higher WTP estimates compared to the Health Star Rating, suggesting that consumers value the more detailed and informative scoring format. Our welfare analysis, based on compensating variation, indicates that introducing evaluative labels leads to substantial increases in consumer welfare, particularly for the Nutrition Score System. The welfare gains were most pronounced among respondents who received an information intervention, highlighting the effectiveness of consumer education in maximizing the perceived value of nutritional labels. The heterogeneity analysis of WTP also revealed that sociodemographic factors, such as education level, significantly influence consumer preferences for FOP labels. These findings extend beyond the Chinese context. Many developing economies experience similar shifts toward packaged foods but differ in label familiarity and nutrition literacy (25, 28). The discrete choice experiment, combined with our information intervention protocol, offers a portable toolkit for quantifying consumers’ WTP for interpretive nutrition labels in various contexts. Applying this methodology across additional emerging markets will enable systematic benchmarking of label performance, uncover population-specific determinants of acceptance, and contribute to a harmonized evidence base for global FOP-labeling policy.

The findings of this study have important policy implications to operationalize evaluative FOP labels in China and other developing markets. First, the government could issue a national standard that mandates a single, easy-to-interpret evaluative format, such as the Nutrition Score System, with precise graphic specifications, nutrient thresholds, and placement rules to ensure uniform presentation across products. Second, a phased rollout should start in large metropolitan supermarkets, where consumer receptivity is highest, then expand to smaller cities and rural counties once supply-chain compliance systems are established and effectiveness metrics have been evaluated. Third, a multi-tiered public education campaign is essential. Mass media spots on television, radio, and social media platforms can raise basic awareness; point-of-sale signage and QR code links on packaging can offer on-demand guidance; and school curricula can integrate label literacy into health and science courses, thereby reaching future consumers early. Fourth, manufacturers and retailers can be encouraged to reformulate products and adopt the label through fiscal incentives such as tax credits or expedited product approvals, while public–private partnerships can fund outreach in lower-income areas. Finally, an inter-agency monitoring framework should track adoption rates, label comprehension, and changes in dietary purchasing patterns, allowing regulators to refine nutrient thresholds and communication strategies over time.

Nevertheless, this study has a couple of limitations. First, the study was conducted in five major cities in China, which may limit the generalizability of the findings to a national level. Although focusing on these cities allowed for an in-depth analysis of consumer preferences for evaluative FOP nutrition labels within different geographic and economic contexts, future research could benefit from incorporating a more extensive and diverse sample across various regions of China. Second, it is essential to recognize that consumer preferences for food products with evaluative FOP nutrition labels can be influenced by factors other than those considered in this study. Attributes, such as freshness and brand play significant roles in consumer decision-making and can interact with FOP labels in shaping preferences and WTP. Future studies should include these product attributes to capture a more holistic view of consumer preferences, thereby improving the understanding of how multiple product features influence consumer choices for FOP labels. Third, similar to many internet surveys conducted in China, our sample contains a modest tilt toward younger and better educated respondents, reflecting the demographic profile of frequent online users (33, 62). While this group offers important insights into urban consumers’ valuation of evaluative FOP labels, it does not capture the full diversity of the national population. Including older age groups, individuals with lower levels of formal education, and residents of rural areas in future data collection would provide a more comprehensive understanding of WTP for interpretive nutrition labeling across China.

## Data Availability

The raw data supporting the conclusions of this article will be made available by the authors without undue reservation.

## Footnotes

^1^Data source: Global Info research. https://globalinforesearch.com.cn/reports/883421/ham-sausage.
