# Supplementary material for: Consumer preferences for evaluative front-of-package nutrition labels: evidence from a choice experiment in China
Source: Front Nutr. 2025 Jul 14;12:1563341. doi: 10.3389/fnut.2025.1563341 (PMC12301347; doi:10.3389/fnut.2025.1563341)
Supplement: Supplementary file 1 [file Data_Sheet_1.docx]

# Appendices:

The evaluative nutrition label is a simplified explanatory tool designed to provide individuals with essential nutrition information. Currently, the United States, Britain, Australia, and other countries utilize various forms of evaluative nutrition labels to present food nutrition information in a more intuitive manner. The primary purpose of adding the evaluative nutrition label is:

(1) to assist consumers in better understanding food nutrition information and making a quick assessment of the nutritional quality of food.

(2) to enable consumers to select nutritious food more judiciously and structure their diets more scientifically.

**Figure A1. Information intervention content**


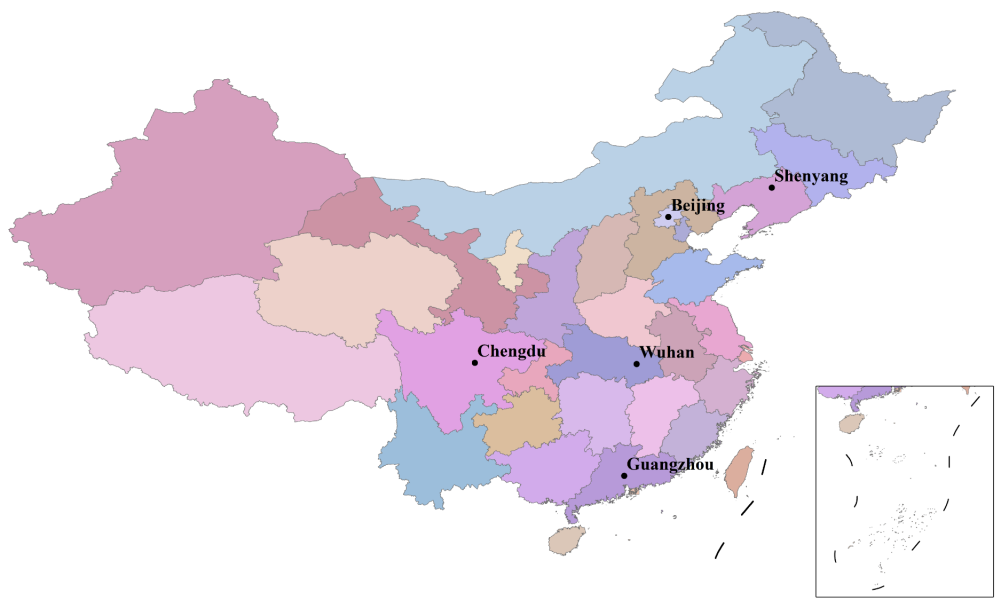


**Figure A2. Geographic distribution of sampled cities**

**Table A1. Estimates of WTP premiums in RPL models**

|  | Pooled | | Information | | | |
| --- | --- | --- | --- | --- | --- | --- |
|  |  |  | No | | Yes | |
|  | WTP | 90% CI | WTP | 90% CI | WTP | 90% CI |
| Star | 1.556 | [0.457, 2.655] | 1.444 | [0.073, 2.816] | 1.831 | [0.061, 3.601] |
|  |  |  |  |  |  |  |
| Score | 1.858 | [0.709, 3.007] | 1.747 | [0.346, 3.148] | 2.119 | [0.248, 3.991] |
|  |  |  |  |  |  |  |
| Contain | 0.266 | [-0.063, 0.594] | 0.151 | [-0.268, 0.569] | 0.395 | [-0.161, 0.950] |
|  |  |  |  |  |  |  |
